# Data to diagnosis: Machine learning-aided prediction of bloodstream infections

**DOI:** 10.6026/973206300212282

**Published:** 2025-08-31

**Authors:** Mallika Sengupta, Sunitaj Parvin, Aditya Kundu, Shiv Sekhar Chatterjee, Ujjala Ghoshal, Sayantan Banerjee, Kaushik Mukhopadhyay

**Affiliations:** 1 Department of Microbiology, All India Institute of Medical Sciences Kalyani, West Bengal, India; 2 Department of Pharmacology, All India Institute of Medical Sciences Kalyani, West Bengal, India

**Keywords:** Bloodstream infection, machine learning, antimicrobial resistance, prediction models

## Abstract

The application of machine learning (ML) algorithms to routine hematological parameters for early prediction of bloodstream infections and to characterize the microbial and antimicrobial resistance (AMR) profile of culture-positive cases is of interest. A retrospective observational study conducted at AIIMS Kalyani between January 2023 and December 2024, in which blood culture results, AST profiles and 16 routine hematological parameters were collected. ML models including Logistic Regression, Decision Tree, Random Forest and SVM were developed using Python. ROC-AUC, accuracy, sensitivity and specificity were computed for model evaluation. *Escherichia coli*, *Klebsiella pneumoniae*, *Staphylococcus aureus* and *Pseudomonas aeruginosa* were the predominant isolates, with high resistance to cephalosporins and beta-lactam-beta-lactamase inhibitor combinations. The Random Forest model showed the highest predictive power (accuracy: 87%, AUC: 0.70) and key predictors included neutrophil count, CRP, and TLC. The ML models offer promising support for early prediction of BSIs and, when coupled with continuous AMR surveillance, can facilitate rapid diagnosis and guide empirical antibiotic therapy, especially in low-resource settings.

## Background:

Bloodstream infections (BSIs) are among the most severe manifestations of infectious diseases, with the potential to rapidly progress to sepsis and septic shock, leading to significant morbidity and mortality worldwide [1]. Bloodstream infections represent a critical healthcare challenge, particularly in hospitalized and immunocompromised patients, where early diagnosis and timely administration of appropriate antimicrobial therapy are essential to reduce adverse clinical outcomes and mortality [2]. However, conventional diagnostic methods, primarily blood cultures, are often limited by delayed turnaround time, suboptimal sensitivity and the inability to detect fastidious organisms, especially in patients who have received prior empirical antibiotic therapy [3]. The epidemiology of BSIs has evolved considerably, with an increasing burden of multidrug-resistant (MDR) pathogens like Gram-negative bacteria such as *Escherichia coli*, *Klebsiella pneumoniae* and *Pseudomonas aeruginosa* have emerged as predominant causes, often exhibiting resistance to beta-lactams, fluoroquinolones, and aminoglycosides [4]. The alarming rise of carbapenem-resistant Enterobacteriaceae (CRE) has further complicated the therapeutic landscape, necessitating a shift in clinical decision-making toward predictive and precision-based diagnostics [5].

In this context, machine learning (ML) has shown promise as a powerful analytical tool capable of identifying patterns in large and complex datasets, as ML algorithms can be trained on routinely available clinical and laboratory parameters to assist in the early prediction of infectious states, including BSIs and by leveraging haematological indices, biochemical markers and vital signs, these models offer the potential to support diagnostic workflows even before microbiological confirmation [6]. Several studies have explored ML applications in infectious disease diagnostics and highlighted the integration of AI in healthcare, particularly its capacity to complement clinical intuition [7]. In the field of sepsis and BSI, ML models have demonstrated moderate to high sensitivity in identifying at-risk patients. Parameters such as neutrophil-to-lymphocyte ratio (NLR), C-reactive protein (CRP), total leukocyte count (TLC) and procalcitonin have been frequently employed in ML frameworks to distinguish bacterial infections from non-infectious causes of systemic inflammation [8]. Despite growing interest, there is a dearth of studies utilizing ML in the Indian context, particularly in tertiary care settings with a high burden of AMR, highlighting a critical gap in understanding how predictive models perform when applied to real-world data from resource-constrained environments. There is a need to bridge that gap by combining routine hematological data, microbiological culture outcomes and ML modeling to build a predictive framework for bloodstream infections. Therefore, it is interest to identify the microbial spectrum and resistance patterns of culture-confirmed BSIs and to evaluate the performance of supervised ML models in predicting culture positivity.

## Methods:

This retrospective observational study was conducted at the Department of Microbiology, All India Institute of Medical Sciences (AIIMS), Kalyani, over a period of two years from January 2023 to December 2024. The study was approved by the Institutional Ethics Committee (Ref. No. IEC/AIIMS/Kalyani/certificate/2024/368 dated 15th November 2024). As the study did not involve any sample collection from the patients directly hence waiver of consent was obtained for the study. Patient identifiers were anonymized and confidentiality of all data was maintained throughout the analysis. Patients of all age groups who presented with clinical suspicion of bloodstream infection and underwent blood culture testing were included. Only the first positive culture episode per patient was considered to avoid duplication. Blood samples were collected aseptically and inoculated into BACT/ALERT FA Plus culture bottles (bioMérieux, France) and incubated in an automated blood culture system. Once flagged positive, subculture was performed on Blood agar and MacConkey agar. Pathogens were identified using standard biochemical tests and the VITEK® 2 Compact system (bioMérieux, France). Antimicrobial susceptibility testing (AST) was conducted using the Kirby-Bauer disc diffusion method on Mueller Hinton Agar and minimum inhibitory concentrations (MICs) were determined for select isolates using broth microdilution as per CLSI 2023 and 2024 guidelines. Results were interpreted as susceptible, intermediate or resistant based on CLSI breakpoints. For ML modeling, demographic and laboratory data including total leukocyte count (TLC), differential counts, hemoglobin, hematocrit, platelets, CRP, and other routine parameters were retrieved from the hospital laboratory information system. Cases were labeled as 'positive' or 'negative' based on blood culture results. Data preprocessing included missing value imputation, normalization, and feature selection. ML models developed included Logistic Regression, Decision Tree, Random Forest, and Support Vector Machine (SVM), implemented using Python's scikit-learn library. Model performance was evaluated using accuracy, sensitivity, specificity, precision, F1 score and the area under the receiver operating characteristic curve (ROC-AUC). Ten-fold cross-validation was employed to avoid overfitting. Feature importance was analyzed to determine the most influential parameters contributing to prediction.

## Results:

A retrospective study was carried out in the Department of Microbiology at a tertiary care center in Eastern India, analyzing data from patients clinically suspected of bloodstream infections over a two-year period (January 1, 2023, to December 31, 2024). A total of 1626 patients suspected of blood stream infection were included in the study. The descriptive summary is given in Table 1 (see PDF). The 133 culture-positive isolates included Enterobacterales (*E. coli*, *Klebsiella pneumoniae*, *Enterobacter spp.*), enteric fever agents (Salmonella Typhi, *S. Paratyphi* A/B), non-fermenting Gram-negative bacilli (*Pseudomonas aeruginosa*, *Acinetobacter baumanii*) and *Staphylococcus aureus*. Less commonly detected organisms included *Stenotrophomonas maltophilia* (8) *Sphingomonas paucimobilis* (5), *Enterococcus spp*. (5), *Candida spp*. (4), *Aeromonas spp*. (3), *Serratia spp*. (3), *Burkholderia cepacia* (3), *Ochrobactrum spp*. (3) and *Ralstonia spp*. (3). *Rhizobactor spp*. was found in 2 samples, while *Streptococcus spp*., *Moraxella ovis*, *Escherichia hermanii*, *Chryseobacterium spp*., *Granulicatella elegans*, *Streptococcus pseudoporcinus*, *Commamonas spp*., *Elizabethkingia spp*., *Leuconostoc spp*. and *Gardenella spp*. were each isolated once. Among the Enterobacterales group comprising *Escherichia coli* (24 isolates), *Klebsiella pneumoniae* (14) and *Enterobacter spp.* (2), notable variations were observed in antimicrobial susceptibility. *E. coli* exhibited the highest sensitivity to amikacin (83.33%), meropenem (79.16%) and piperacillin-tazobactam (75%), while ciprofloxacin and levofloxacin showed lower efficacy, with sensitivities of 33.33% and 45.83%, respectively.

The enteric fever group, including *S. Typhi* (8 isolates), *S. Paratyphi* A (4) and *S. Paratyphi* B (1), demonstrated excellent susceptibility (100%) sensitive to third generation cephalosporins, carbapenems, chloramphenicol and cotrimoxazole. The resistance to fluoroquinolones was high 66.7%. The non-fermenting Gram-negative bacilli, *Pseudomonas aeruginosa* (12 isolates) and Acinetobacter baumannii (7), showed contrasting resistance patterns. Pseudomonas retained high sensitivity to carbapenems (91.66%) and piperacillin-tazobactam (83.33%) and aminoglycosides (91.66%), while fluoroquinolone sensitivity was notably lower (25%). In stark contrast, Acinetobacter demonstrated marked resistance, with sensitivities not exceeding 42.85% for any antibiotic. Most agents showed less than 30% efficacy with meropenem (42.85%) and aminoglycosides (28.57%). For *Staphylococcus aureus* (15 isolates), the highest susceptibility was observed with vancomycin and linezolid (100%), followed closely by doxycycline (86.66%). However, there was 46.6% methicillin Resistant *Staphylococcus aureus* (MRSA) and significant resistance to fluoroquinolones (ciprofloxacin 86.7%, levofloxacin 66.7%) and co-trimoxazole (53.3%). Machine learning performance was evaluated using eight classifiers. The Random Forest model achieved the highest accuracy (87.6%) and AUC (0.70), with a sensitivity of 36.4% and specificity of 98.1% (Table 2 - see PDF and Figure 1 - see PDF). Of 260 patients used for ML modeling, 44 showed culture positivity and 216 were negative. The Random Forest, LDA and Logistic Regression models performed reasonably well; however, ensemble methods provided more balanced accuracy and NPV for screening use as shown in Table 2 (see PDF).

## Discussion:

Several studies worldwide have explored the application of Machine Learning (ML) to predict bloodstream infections and related outcomes, with promising results across diverse clinical settings. These studies have utilized a range of input variables-from routine laboratory parameters to complex electronic health record (EHR) datasets-to improve the early detection of bacteremia and sepsis. Despite this growing body of evidence, there are only few published studies from India which have evaluated the role of ML in predicting positive blood cultures or bloodstream infections. Given India's high burden of antimicrobial resistance (AMR) and variability in healthcare infrastructure, locally derived predictive models are crucial to ensure clinical relevance and implementation feasibility. The studies summarized below provide valuable insights and a foundation for developing context-specific ML tools for septicemia. A study by Giannini *et al.* developed a machine learning algorithm using electronic health record data including digital patient information *i.e.*, vitals, laboratory parameters and clinical notes to predict severe sepsis and septic shock. The algorithm showed 76% sensitivity and 82% specificity [9]. Xu *et al.* from China, used real-world data from the Medical Information Mart for Intensive Care IV (MIMIC-IV) database to cluster 2,339 sepsis patients based on blood culture results. Machine learning analysis revealed distinct survival and severity patterns, suggesting innovative prognostic and therapeutic strategies. Using unsupervised K-means clustering based on blood culture profiles, they identified five distinct bacterial infection clusters. Each cluster showed significant variation in disease severity (measured by GCS, SOFA, SAPSII and SIRS scores) and survival outcomes. The 28-day survival differences were statistically significant (p = 4.4e-5), while the 7-day survival curves were less differentiated [10]. Lien *et al.* Taiwan and developed a machine learning model using only complete blood count and differential leukocyte count data to predict bacteremia. The model achieved an AUC of 0.802, outperforming CRP and procalcitonin tests, offering rapid, cost-effective and comparable prognostic capability for clinical use [11]. Bedoya *et al.* at Duke University developed a novel deep learning model - Multi-output Gaussian Process coupled with a Recurrent Neural Network (MGP - RNN) to detect sepsis early using electronic health records. Validated on over 80,000 encounters, it achieved an AUROC of 0.88 and predicted sepsis a median of 5 hours in advance, outperforming clinical scores like SIRS, qSOFA, NEWS and traditional machine learning models [12].

Dhungana *et al.* at Mayo Clinic, USA developed a supervised machine learning-based computable phenotype to identify sepsis and septic shock using ICU electronic medical records. Based on SOFA scores, cultures and lactate and vasopressor use, it achieved 100% sensitivity and specificity in validation, offering a fast, reliable alternative to manual chart review [13]. Chang *et al.* Taiwan and developed a machine learning model using complete blood count (CBC), differential count (DC) and cell population data (CPD) to predict bacteremia. Validated across three hospitals, the CatBoost model achieved AUROCs up to 0.847. Key features included lymphocyte conductivity, neutrophil-to-lymphocyte ratio and nucleated RBCs [14]. Stocker *et al.* Switzerland, conducted a secondary analysis of the Neonatal Procalcitonin Intervention Study (NeoPInS) to develop a machine learning model for predicting neonatal early-onset sepsis. Using a random forest algorithm on data from 1,685 neonates across multiple international centers, they found that biomarkers particularly CRP and white blood cell count (WBC) were far more predictive than clinical signs or risk factors. The model achieved an AUROC of 83.4% and an AUPRC of 28.4%, offering a potential tool to reduce unnecessary antibiotic use in neonates [15]. Mahmoud *et al.* Riyadh and developed machine learning models to predict bacteremia using 21,073 blood cultures. Neural networks achieved 89% specificity but low sensitivity. Key predictors included central line presence, hospital stay >16 days and lactic acid >2 mmol/L. SIRS and qSOFA showed limited utility [16].

Kainth *et al.* conducted a systematic review evaluating Machine Learning (ML) models for diagnosing neonatal sepsis. Screening 5008 records, they included 19 studies (15,984 participants) involving 76 ML models predominantly using random forest algorithms. Most models incorporated birth weight and gestational age as predictors, though none underwent external validation. Pooled sensitivity and specificity were 0.87 and 0.89, respectively, with an AUC of 0.94, indicating strong diagnostic performance. However, risk of bias was high in 18 studies and most were based in high- or upper-middle-income countries [17]. Vijayakumar *et al.* developed and validated an explainable AI system for early prediction of blood culture positivity in neutropenic leukemia patients undergoing chemotherapy (N = 110). Recognizing the critical delay in traditional blood culture results and the risks of broad-spectrum antibiotic overuse, the model utilized readily available hematological and clinical parameters to predict bacterial growth 2-5 days in advance. The best-performing model achieved an accuracy and F1 score of 78%, while predictions specific to gram-negative bacteria reached 63% for both metrics [18]. Zhang and colleagues developed a machine learning model using routine laboratory parameters to distinguish between Gram-positive and Gram-negative bacteremia, achieving the best performance with a Random Forest classifier (AUC = 0.768, sensitivity = 75.20%, specificity = 63.79%) [19]. Murri *et al.* developed a machine learning-based model using multivariate logistic regression to predict the risk of hospital-acquired bloodstream infections, achieving a validation AUROC of 0.74 and enabling classification into low-, medium-, and high-risk groups. The model demonstrated strong negative predictive value (NPV = 0.82), suggesting its utility in antibiotic stewardship by potentially reducing unnecessary antibiotic use in up to 31.1% of low-risk patients [20]. The current study demonstrated that machine learning algorithms, particularly Random Forest, can offer valuable support in the early prediction of bloodstream infections (BSIs) using routine hematological parameters. The overall model performance with accuracy of 87.6%, AUC of 0.70 and a specificity of 98.1% highlights the potential utility of ML in diagnostic triage. However, sensitivity was modest (36.4%), reflecting the challenge of capturing subtle early infection signals, especially with limited positive cases. Key predictive features, including neutrophil count, CRP and total leukocyte count, align with established inflammatory markers in BSIs.

The antimicrobial resistance patterns identified were concerning. While *E. coli* and *Pseudomonas aeruginosa* retained some sensitivity to carbapenems and aminoglycosides, widespread resistance to cephalosporins and beta-lactamase inhibitors underlines the urgency for stewardship efforts. Notably, Acinetobacter exhibited high resistance across all antibiotics. The integration of microbial profiling and ML modeling in this study provides a dual benefit: real-time risk prediction and enhanced antimicrobial guidance. This is particularly relevant in low-resource, high-AMR settings like India, where laboratory confirmation is often delayed. The model offers a promising step toward precision diagnostics tailored to regional epidemiology. This study has several limitations. First, it is a single-center, retrospective analysis, limiting generalizability. Second, the dataset had class imbalance, with relatively few culture-positive cases having complete dataset, potentially affecting model sensitivity. Third, the study excluded patients with incomplete data, which may introduce selection bias. Fourth, performance metrics like sensitivity were modest, which could hinder clinical deployment in isolation. Finally, the model was not externally validated on independent datasets, making its robustness across different populations uncertain. Further multicenter, prospective studies with larger datasets and external validation are necessary to confirm these findings and improve model performance.

## Conclusion:

The feasibility of using machine learning models, especially Random Forest, to predict bloodstream infections based on routine hematological parameters is shown. Despite modest sensitivity, the high specificity and reasonable accuracy suggest its potential utility as a screening tool to aid early clinical decisions. When integrated with microbiological data and antimicrobial resistance profiles, ML-driven prediction can enhance empirical antibiotic selection and improve patient outcomes. Broader validation and inclusion of clinical features could improve sensitivity and support its adoption in real-world settings.

## Declaration:

## Funding:

None

## Publication:

None

## Pre-Print:

Not published

## Ethics approval:

The study was approved by the Institutional Ethics Committee, All India Institute of Medical Sciences, Kalyani (Ref. No.: IEC/AIIMS/Kalyani/certificate/2024/368 dated 15th November 2024).
